# Supplementary material for: scNMT-seq enables joint profiling of chromatin accessibility DNA methylation and transcription in single cells
Source: Nat Commun. 2018 Feb 22;9:781. doi: 10.1038/s41467-018-03149-4 (PMC5823944; doi:10.1038/s41467-018-03149-4)
Supplement: Supplementary file 1 — Supplementary Information [file 41467_2018_3149_MOESM1_ESM.pdf]

scNMT-seq enables joint profiling of chromatin accessibility DNA methylation and transcription in single cells

Stephen J. Clark et al

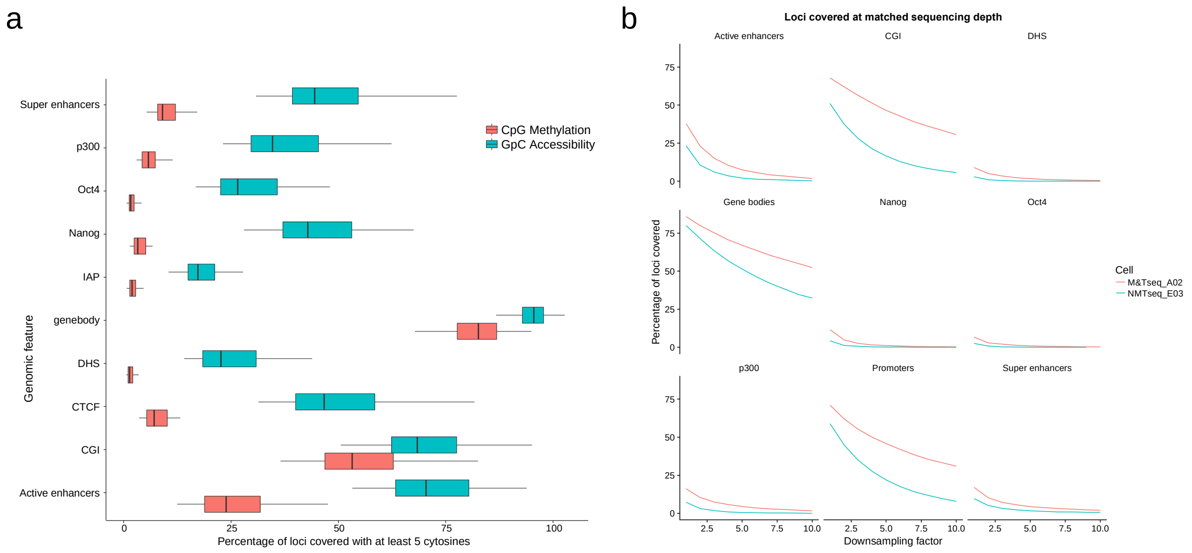

Supplementary figure 1. Empirical coverage of scNMT-seq applied to 61 mouse ES cells. (a) Distribution of the percentage of loci covered by at least 5 cytosines across 61 cells, considering different genomic contexts. Boxes display median coverage and the first and third quartile, whiskers show 1.5 x the interquartile range above and below the box. **(b)** Effect of reduced sequencing depth on coverage – number of loci covered by at least 5 cytosines, comparing data from two cells sequenced at equivalent depth, however using alternative protocols (M&T-seq A02 = 3.41M uniquely aligned reads, NMT-seq E03 = 3.43M uniquely aligned reads). Shown is the coverage of loci as a function of increasing down sampling factors (down to $1/10^{th}$ of the CpG coverage in intervals of $1/10^{th}$).

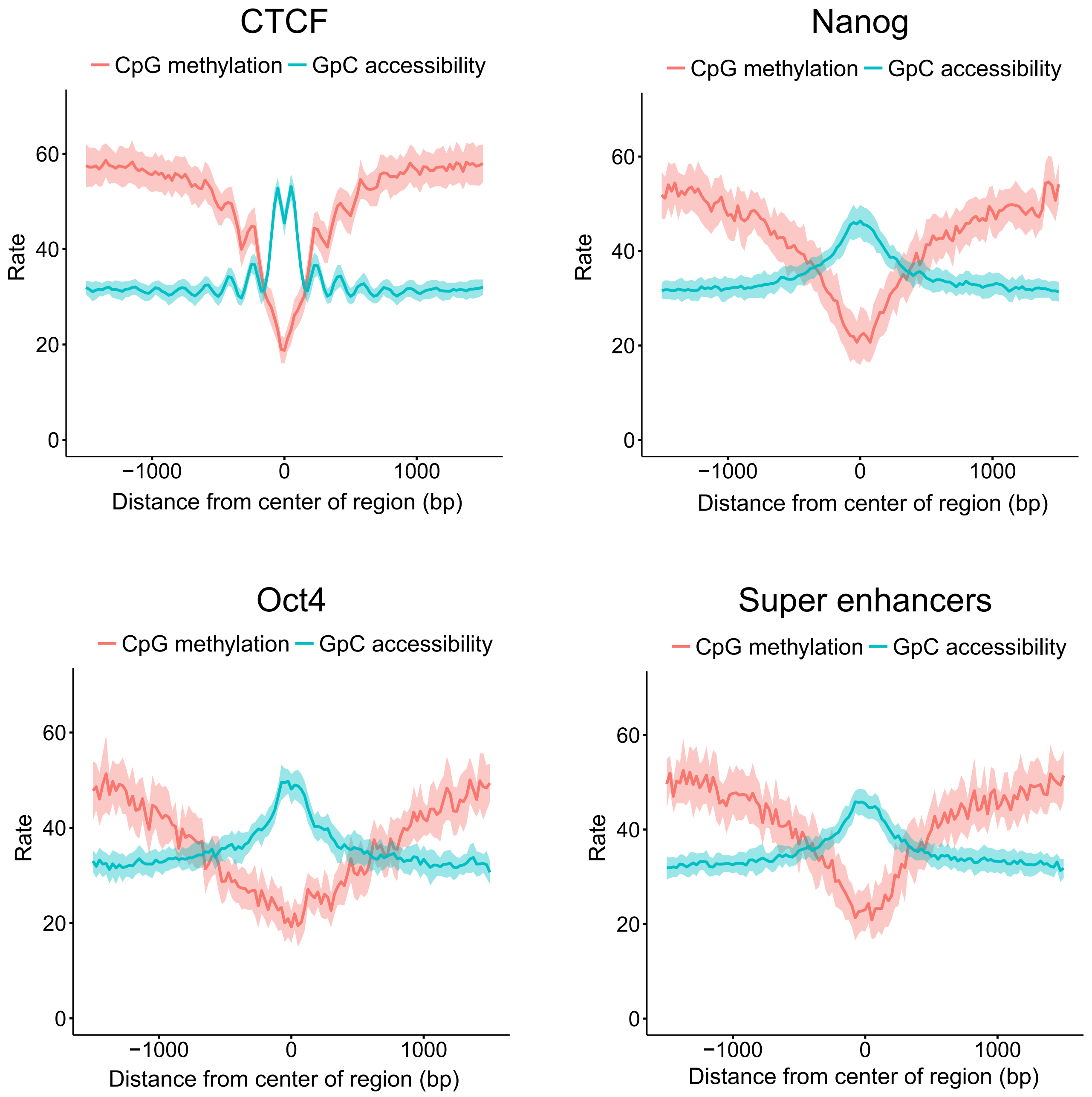

Supplementary figure 2. Accessibility and methylation profiles in regulatory genomic contexts. Shown are running averages of the CpG methylation (red) and the GpC accessibility (blue) in consecutive non-overlapping 50bp windows, pooling information from all cells and all genomic elements in different regulatory contexts. Solid line displays the mean across all cells and loci and shading displays the corresponding standard deviation.

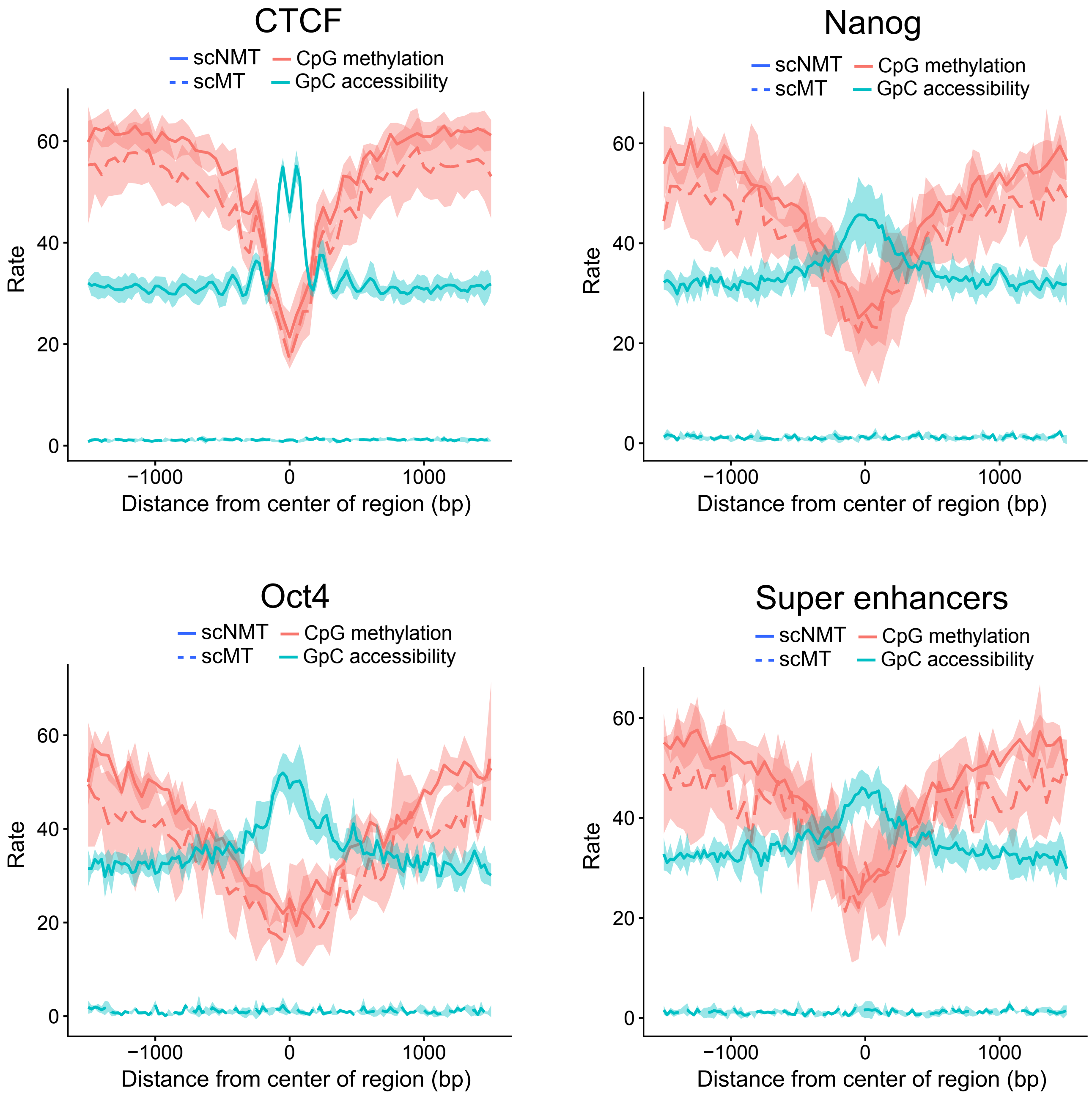

Supplementary figure 3. Accessibility and methylation profiles at regulatory genomic contexts, comparing scNMT-seq to scMT-seq control cells. Shown are running averages of the CpG methylation (red) and the GpC accessibility (blue) in consecutive non-overlapping 50bp windows, pooling information from all cells and all genomic elements in different regulatory contexts. Solid line displays the mean across all cells and loci and shading displays the corresponding standard deviation. Profiles with solid lines are calculated from scNMT-seq cells and profiles with dashed lines are calculated from the 3 scMT control cells (no GpC methyltransferase enzyme treatment). This result shows that endogenous GpC methylation levels are low and therefore do not affect the accessibility data.

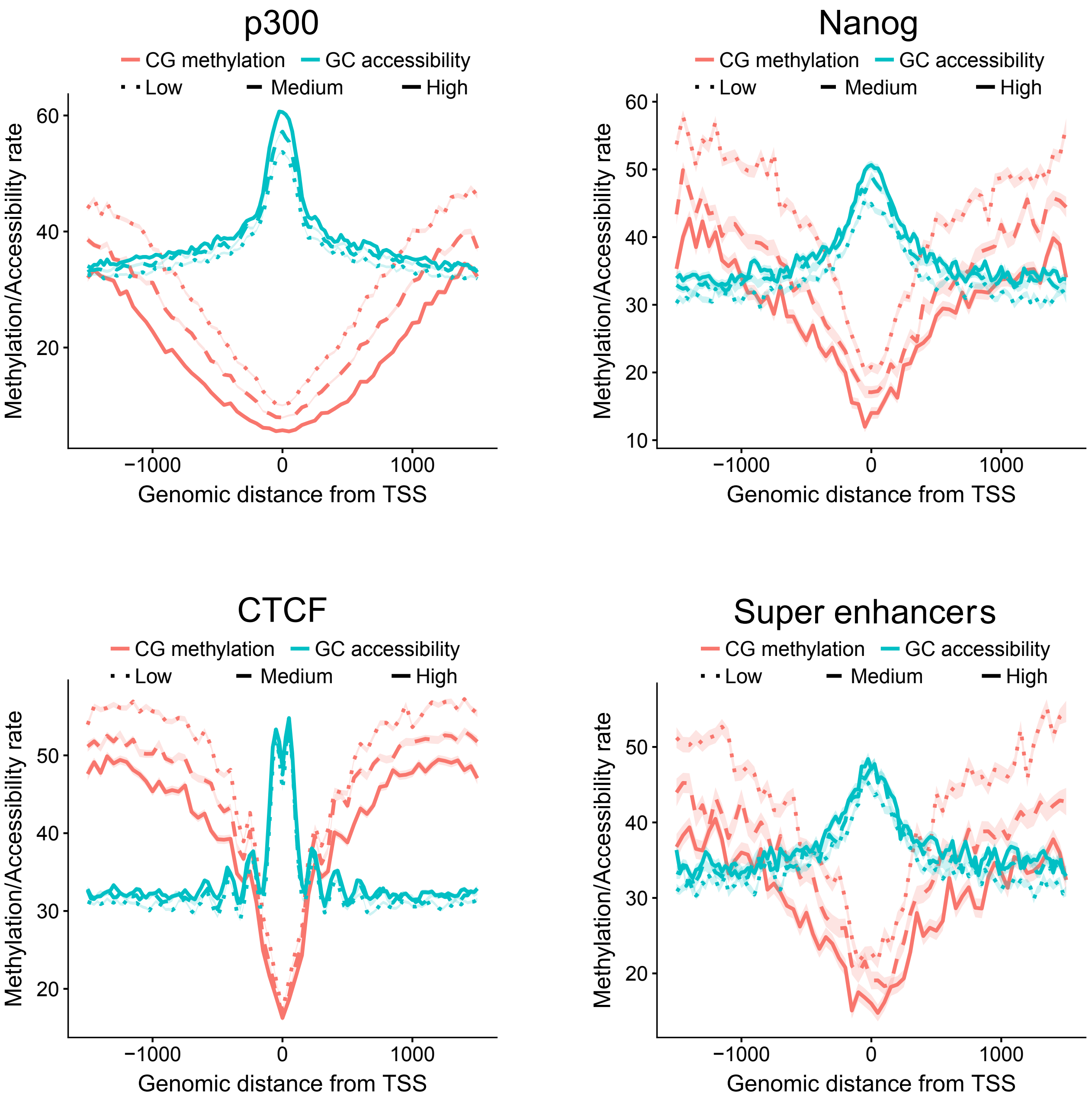

Supplementary figure 4. Accessibility (blue) and methylation (red) profiles at regulatory genomic contexts, stratified by expression of the nearest gene. Shown are local GpC accessibility and CpG methylation profiles for different genomic contexts. Features are stratified by average expression level of the corresponding gene (log normalised counts less than 2 (low), between 2 and 6 (medium) and higher than 5 (high). The profile is generated by computing a running average across all cells and loci in 50bp windows.

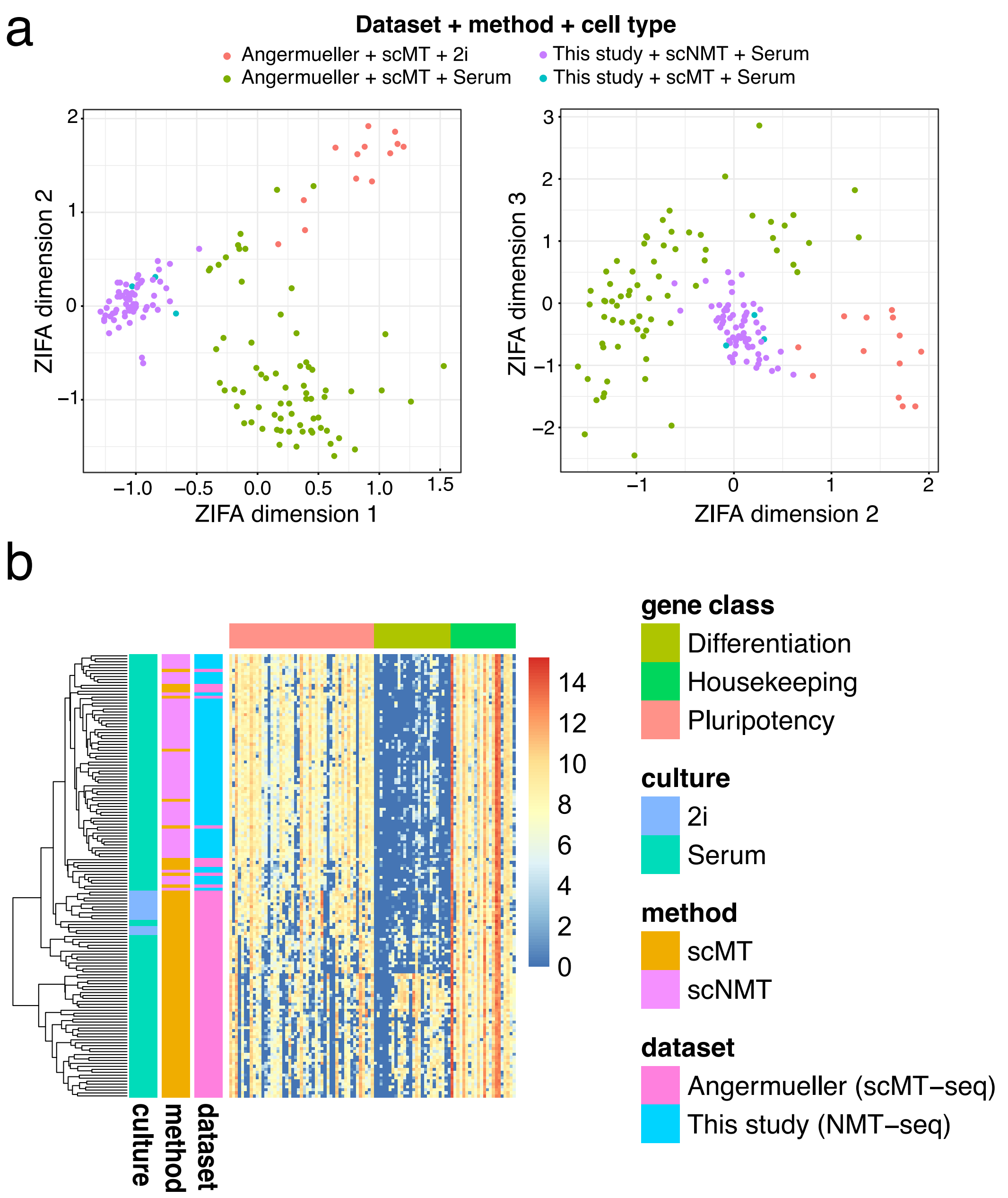

Supplementary figure 5. Comparison of RNA-seq data to previously published data from Angermueller *et al* 2016[1]. (a) ZIFA dimensionality reduction[2] highlighting culture conditions and method used (scNMT-seq or scMTseq). **(b)** Heatmap showing expression of three gene sets: pluripotency genes that have previously been used to classify ESCs as more or less pluripotent[3], differentiation marker genes and housekeeping genes as a control. Serum cells processed in our previous study[1] have a higher degree of transcriptional heterogeneity. This is not likely to be due to protocol differences, since the scMT-seq control cells do not cluster apart from scNMT-seq cells. We suggest that it may reflect differences in the cell lines used for the two studies (male E14 previously versus female EL16 used here). In particular, serum cells in this study appear to belong entirely to a fairly homogeneous sub-population with pluripotency levels closer to the 2i cells from Angermueller *at al*[1].

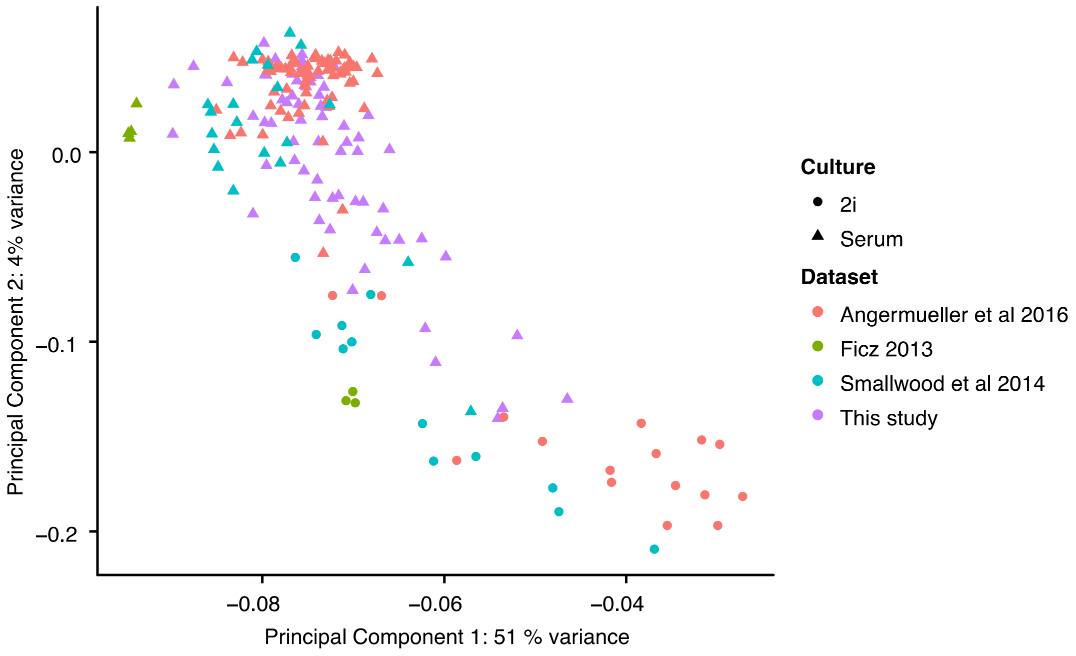

Supplementary figure 6. Comparison of scNMT-seq to published bulk and single-cell methylation profiles. PCA of gene body methylation (all genes) comparing scNMT-seq to published datasets of serum and 2i grown ESCs. Missing sites were imputed using the average methylation rate across cells at a given locus. Serum cells processed in this study overlap with 2i cells in previously published data, which is consistent with the observed clustering of RNA-seq data (Supplementary Fig. 2), indicating that the population of cells considered in this study was more pluripotent than cells in Angermueller et al[1]. These differences likely reflect variation in the cell lines used (male E14 versus female EL16; see Supplementary Fig. 2). Female ESCs are reported to have lower global methylation levels[4] and we find a mean global methylation level of 61% versus 78% in E14 cells[1].

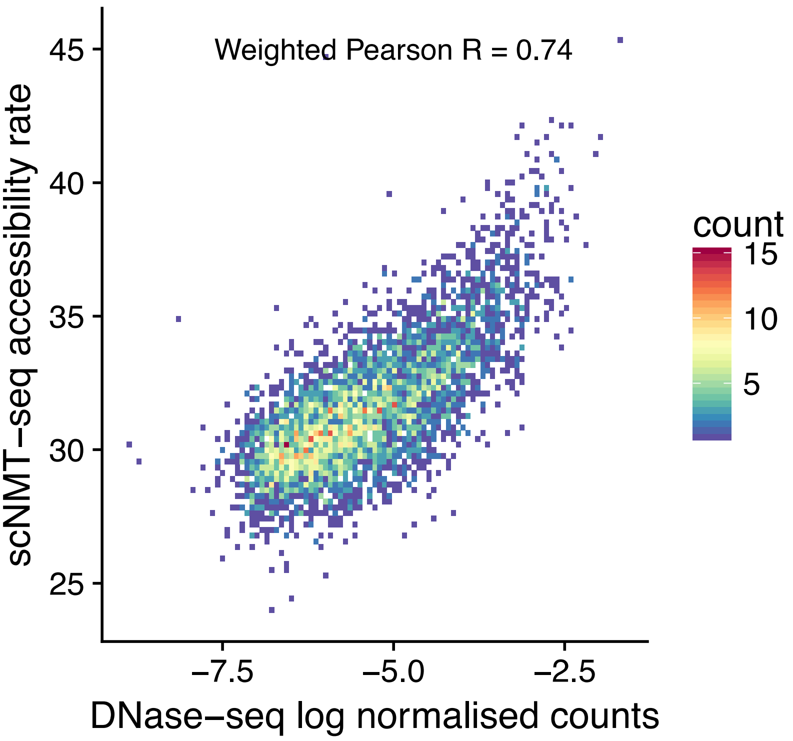

Supplementary figure 7. Scatter plot of GpC accessibility data and published DNase-seq in random 10kb windows. GpC accessibility data show mean GpC methylation rates across all cells in 100,000 random 10kb windows. DNase-seq is log2 reads per bp within the same windows. Pearson correlation coefficient was calculated using a weighting of the GpC coverage (number of observations in the 10kb window), thereby accounting for variation in the coverage of scNMT-seq data, which is sparse (61 cells at ~15% genome-wide coverage each) and dependent on GpC density.

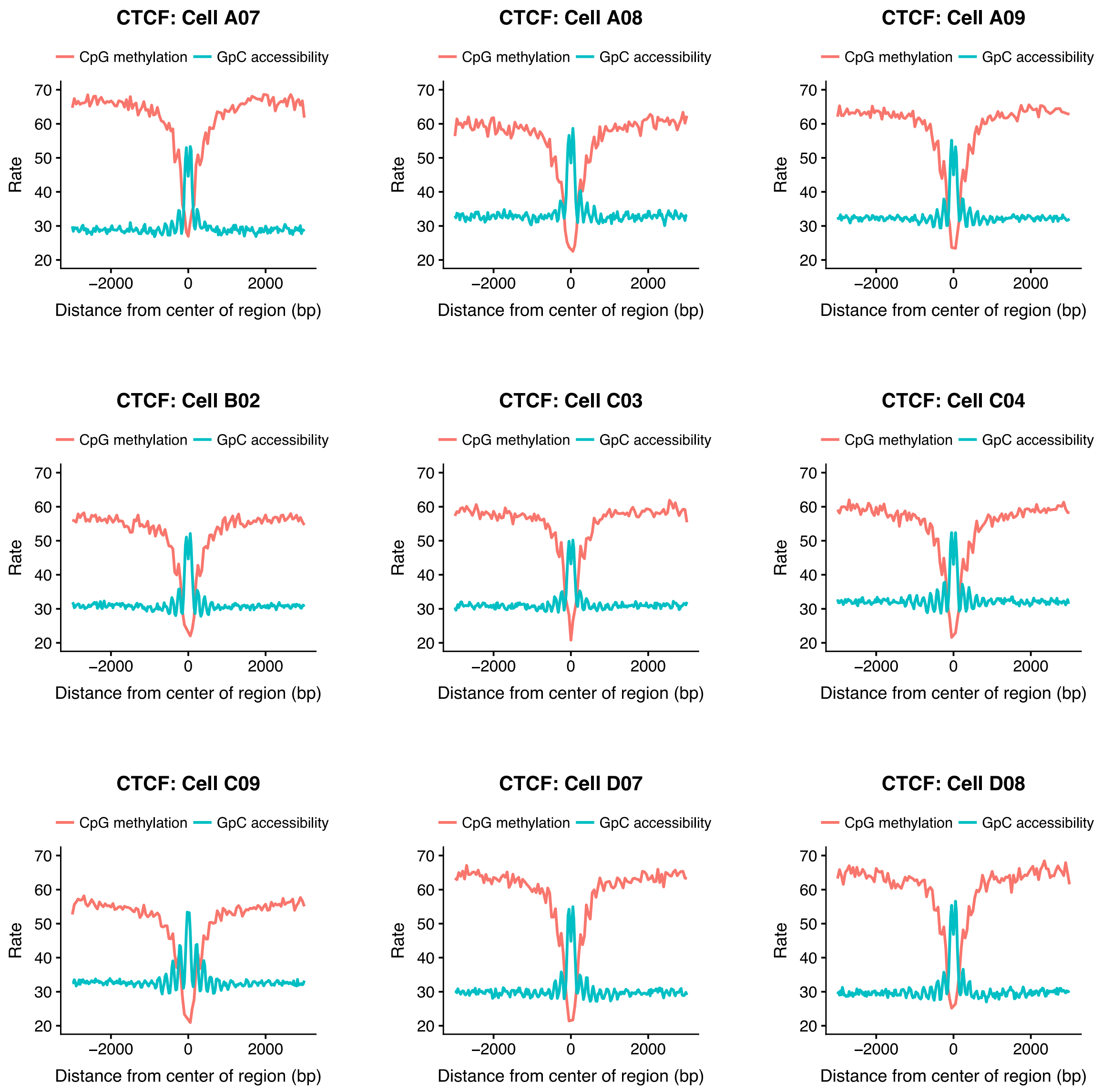

Supplementary figure 8. Accessibility and methylation profiles in regulatory genomic contexts in single cells. Shown are running averages of the CpG methylation (red) and the GpC accessibility (blue) in consecutive non-overlapping 50bp windows, pooling information from all genomic elements in different regulatory contexts. Solid line displays the mean across all loci and shading displays the corresponding standard deviation. This result shows that the scNMT-seq recovers, in single cells, the methylation and accessible profiles obtained with pseudobulked data shown in Supplementary Fig. 2.

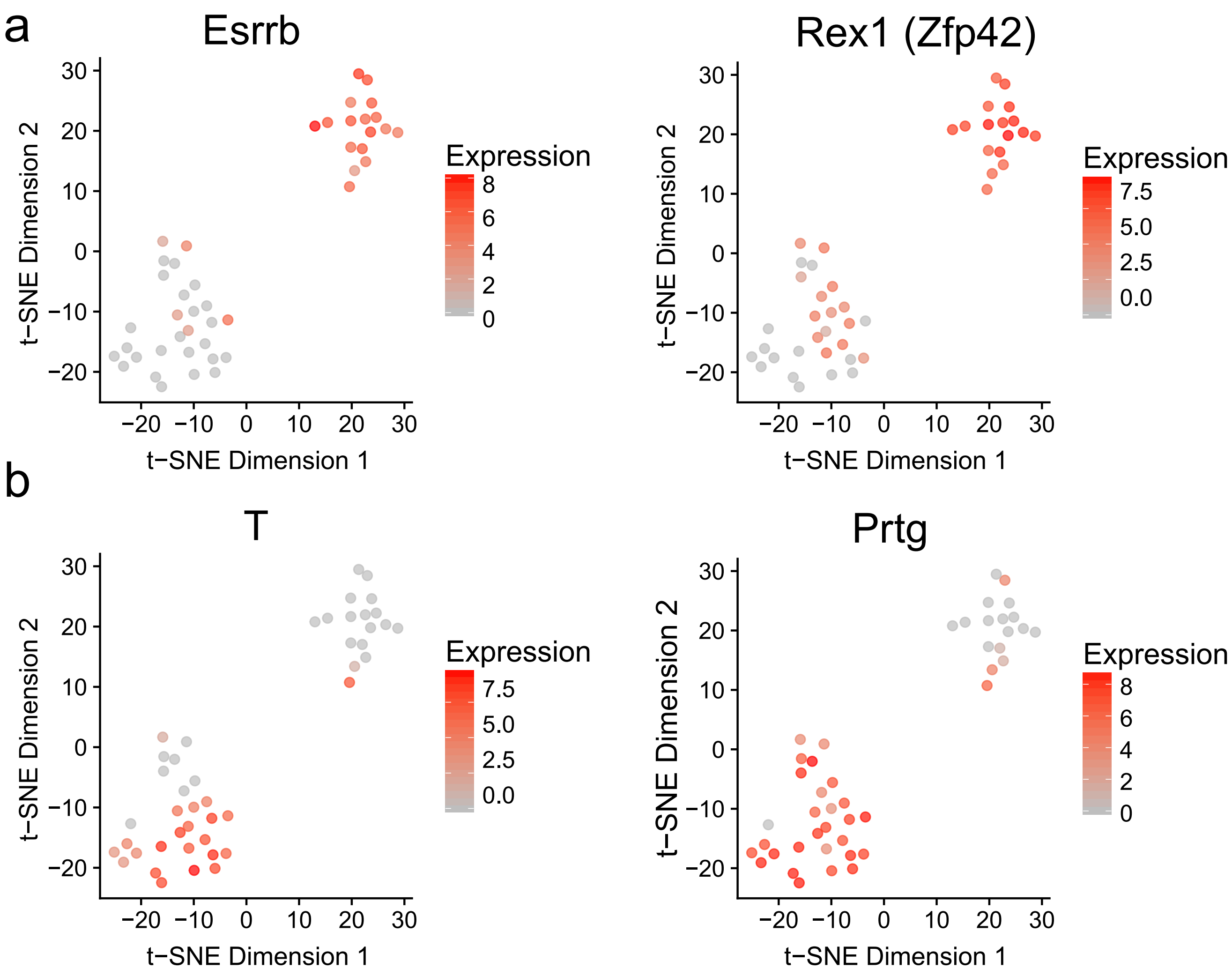

Supplementary figure 9. Visualisation of RNA-seq profiles of 43 embryoid body cells. Shown are bivariate visualizations using t-SNE, with expression profiles of canonical (a) pluripotency and (b) differentiation markers genes, overlaid in colour. Cells cluster into two main populations, which we subsequently labelled as pluripotent (high expression of pluripotency genes) and differentiated (low expression levels of pluripotency genes).

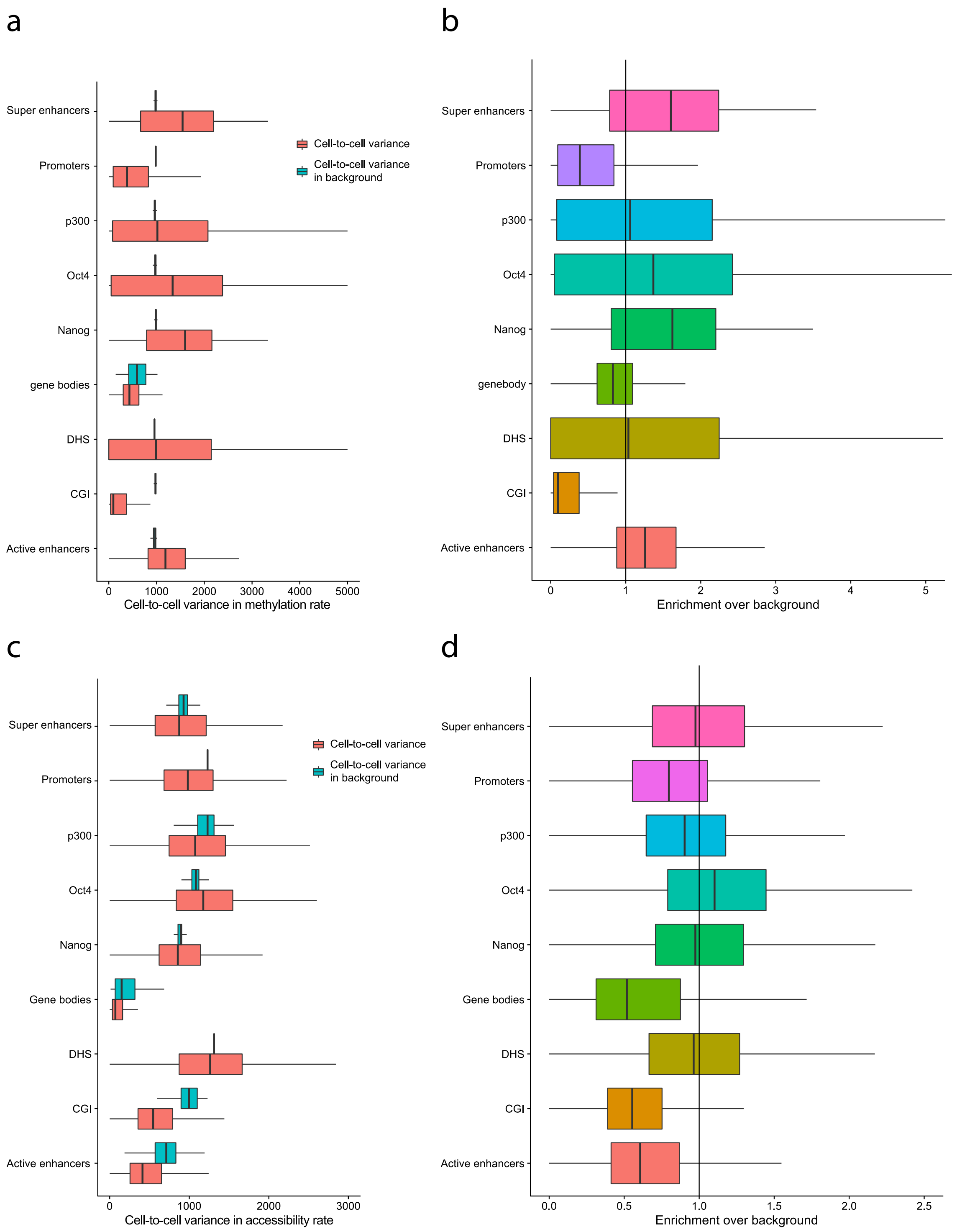

Supplementary figure 10. Cell-to-cell variance in methylation and accessibility rates at different genomic contexts in EB cells. (a) Cell-to-cell variance in methylation rate in selected genomic contexts (red) and in random regions of matched size (blue). **(b)** Enrichment of methylation variance in the contexts as in **a** compared to random background regions, matched for size. The background variance is the mean cell-to-cell variance from 10,000 random loci, calculated for each size. **(c)** Cell-to-cell variance in accessibility rate in selected genomic contexts (red) and cell-to-cell variance in random regions of matched size (blue). **(d)** Enrichment of accessibility variance above background. For all plots, boxes display medians and the first and third quartiles, whiskers show 1.5 x the interquartile range.

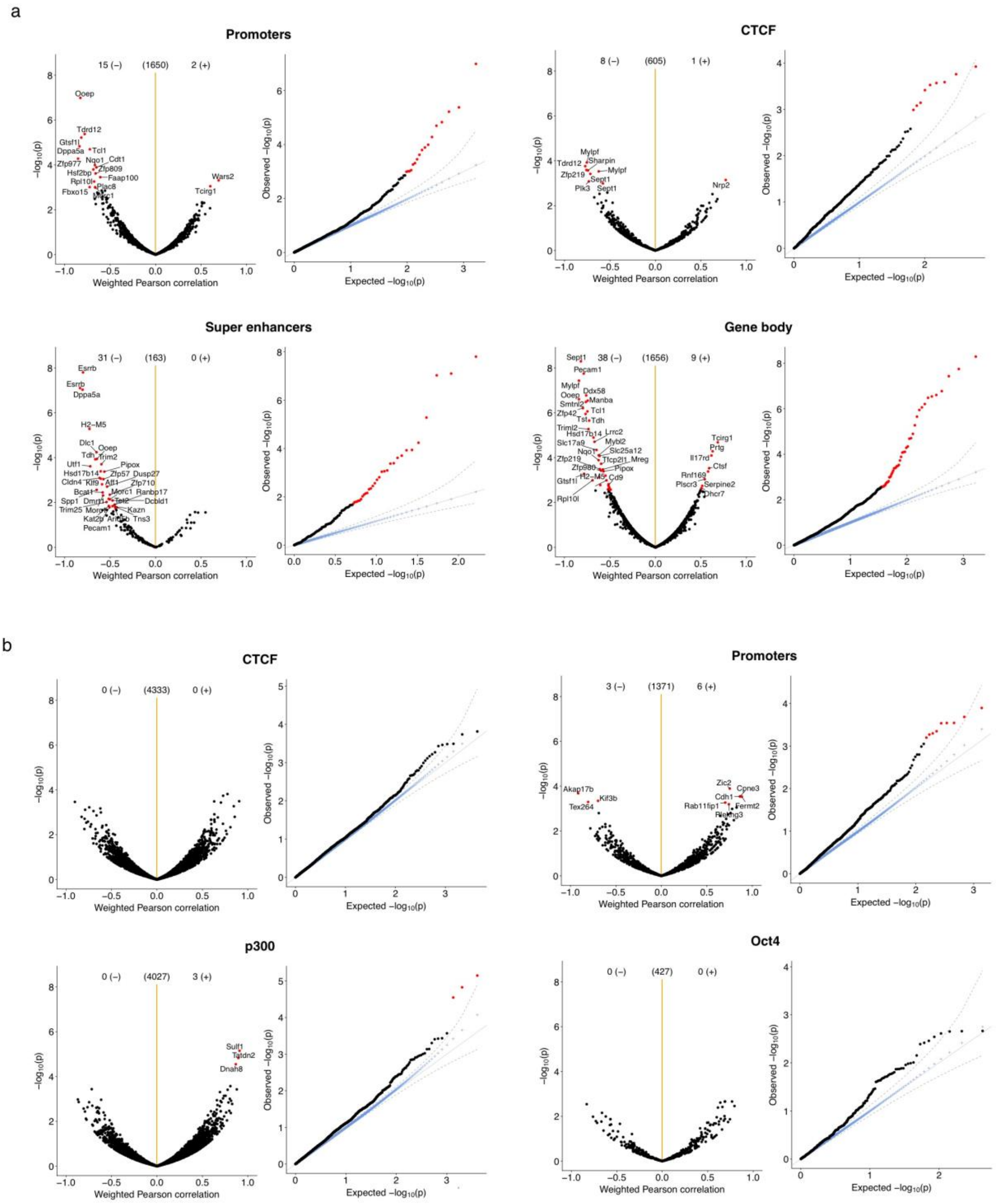
a
Promoters
15 (−)
(1650)
2 (+)
Ooep
Tdrd12
Gtsf1l
Dppa5a
Tcl1
Zfp977
Nqo1
Cdt1
Zfp809
Hsf2bp
Faap100
Rpl10l
Plac8
Fbxo15
Wars2
Tcirg1
CTCF
8 (−)
(605)
1 (+)
Mylpf
Tdrd12
Sharpin
Zfp219
Sept1
Plk3
Nrp2
Super enhancers
31 (−)
(163)
0 (+)
Esrrb
Dppa5a
H2−M5
Dlc1
Ooep
Tdh
Trim2
Utf1
Pipox
Hsd17b14
Zfp57
Dusp27
Cldn4
Klf9
Zfp710
Bcat1
Morc1
Ranbp17
Spp1
Dcbld1
Trim25
Kazn
Kat2b
Tns3
Pecam1
Gene body
38 (−)
(1656)
9 (+)
Sept1
Pecam1
Mylpf
Ddx58
Ooep
Manba
Smtnl2
Zfp42
Tcl1
Tst
Tdh
Triml2
Hsd17b14
Lrrc2
Slc17a9
Mybl2
Slc25a12
Nqo1
Tfcp2l1
Mreg
Zfp219
Zfp980
Pipox
H2−M5
Cd9
Gtsf1l
Rpl10l
Tcirg1
Prtg
Il17rd
Ctsf
Rnf169
Plscr3
Serpine2
Dhcr7
b
CTCF
0 (−)
(4333)
0 (+)
Promoters
3 (−)
(1371)
6 (+)
Akap17b
Kif3b
Tex264
Zic2
Cpne3
Cdh1
Rab11fip1
Fermt2
p300
0 (−)
(4027)
3 (+)
Sulf1
Dnah8
Oct4
0 (−)
(427)
0 (+)
Weighted Pearson correlation
$-\log_{10}(p)$
Observed $-\log_{10}(p)$
Expected $-\log_{10}(p)$

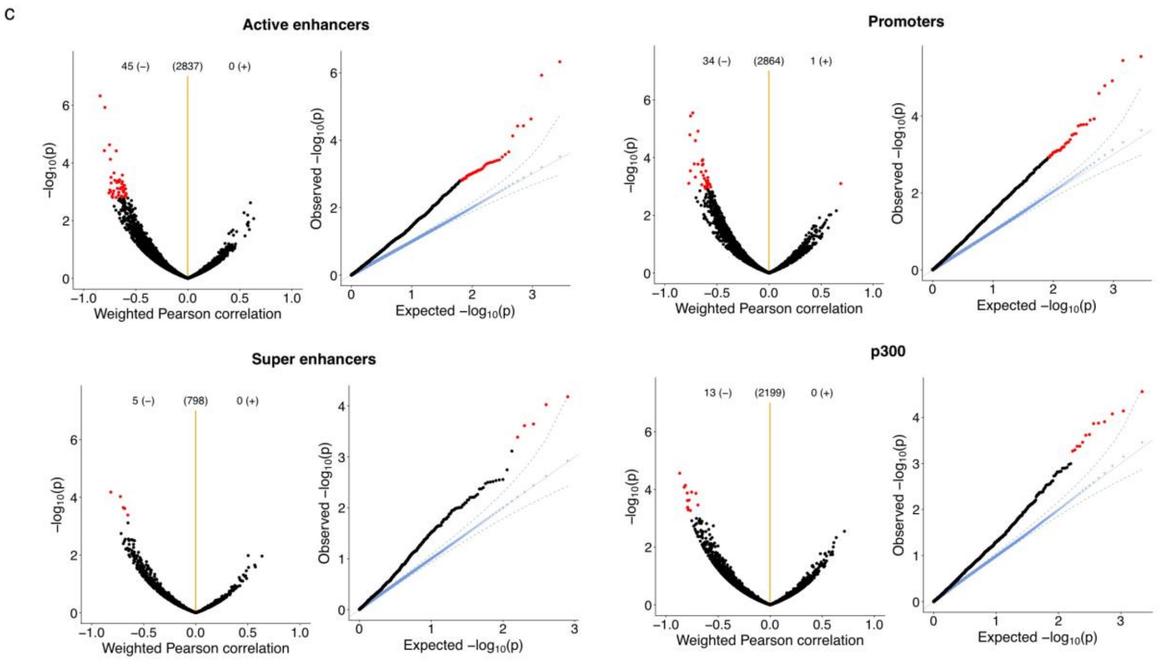

Supplementary figure 11. Association tests between molecular layers in selected genomic contexts. Shown are correlation analysis across cells (one test per loci) between (a) CpG Methylation and RNA expression, (b) GpC accessibility and RNA expression and (c) CpG methylation and GpC accessibility. Volcano plots display Pearson correlation coefficients and adjusted p-values (Benjamini-Hochberg correction). The orange vertical lines show the position of r=0. Red dots denote features that pass threshold of statistical significance (FDR=10%). Q-Q plots show the distribution of observed p-values (black and red dots), the uniform distribution (grey lines, with solid line showing the mean and the dashed line showing the 95% confidence interval) and p-values obtained after 100 permutations of both features and samples (blue crosses)

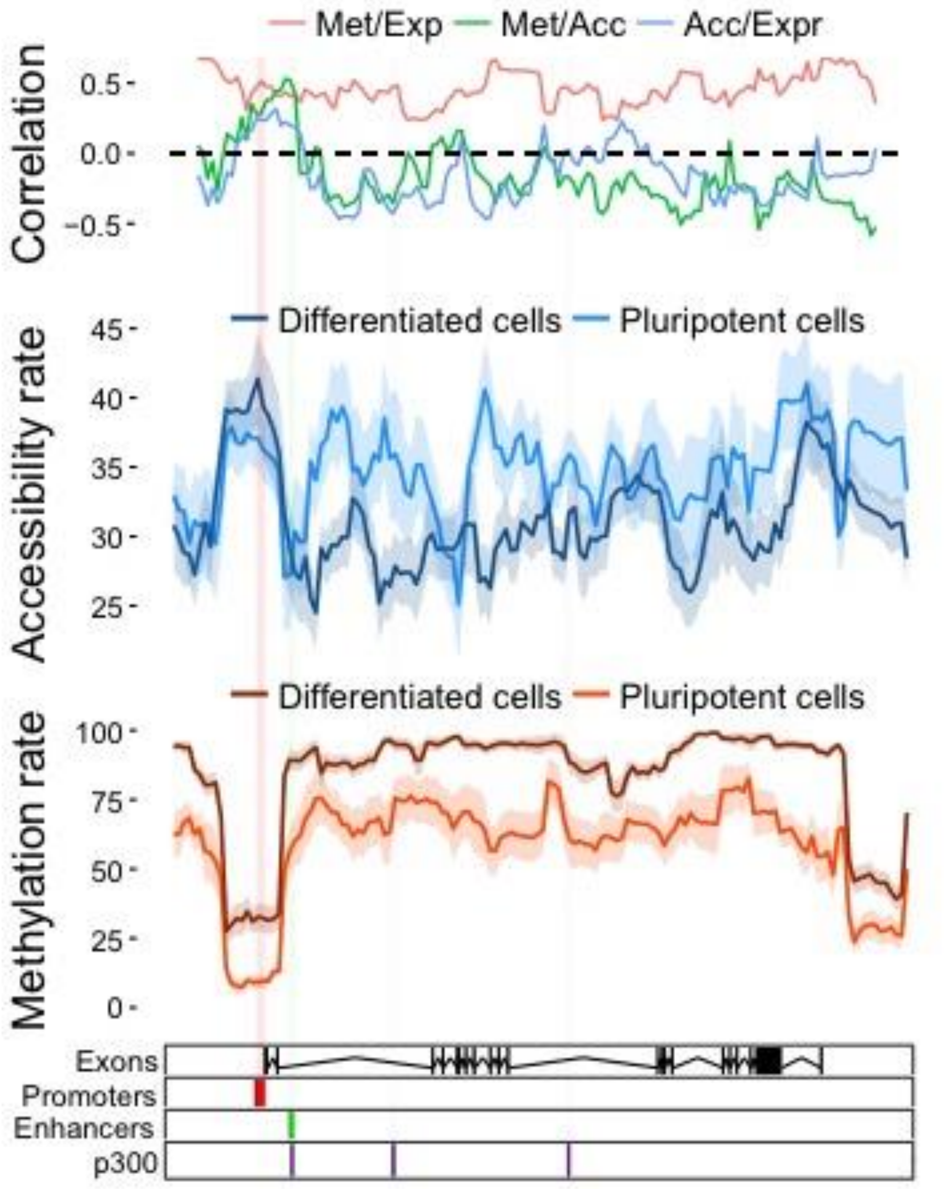

Supplementary figure 12. Zoom-in view within the gene locus of *Prtg* for embryoid body data. Shown from top to bottom are: Pairwise Pearson correlation coefficients between each pair of the three layers (Met, methylation; Acc, accessibility; Expr, expression). Accessibility (blue) and methylation (red) profiles are shown separately for the pluripotent and differentiated sub-populations; mean rates (solid line) and standard deviation (shade) were calculated across cells using a running window of 10kb with a step size of 1000bp; Track with genomic annotations, highlighting the position of several regulatory elements: promoters, super enhancers, and p300 binding sites.

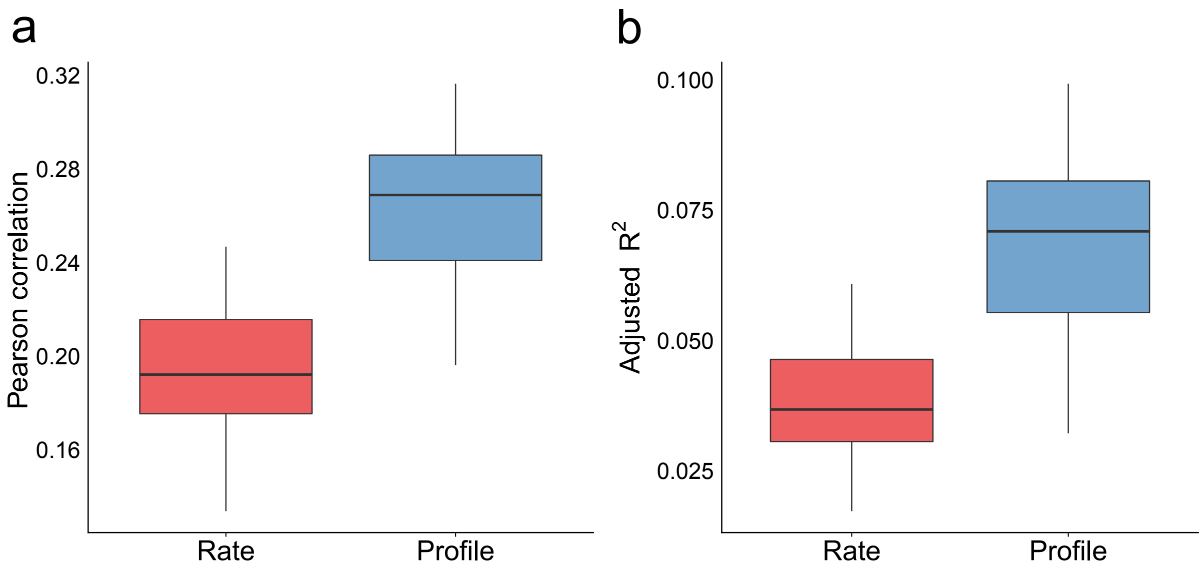

Supplementary Figure 13. Accessibility profiles predict gene expression more accurately than accessibility rates. Shown are correlation coefficients between observed gene expression levels and predicted gene expression levels using accessibility rates (red) and accessibility profiles (blue). Correlations are computed across genes, so each data point is one cell. The plots show (a) the Pearson correlation coefficient and (b) the R^2 adjusted to correct for the increased amount of parameters in the model. For both plots, boxes display medians and the first and third quartiles, whiskers show 1.5 x the interquartile range.

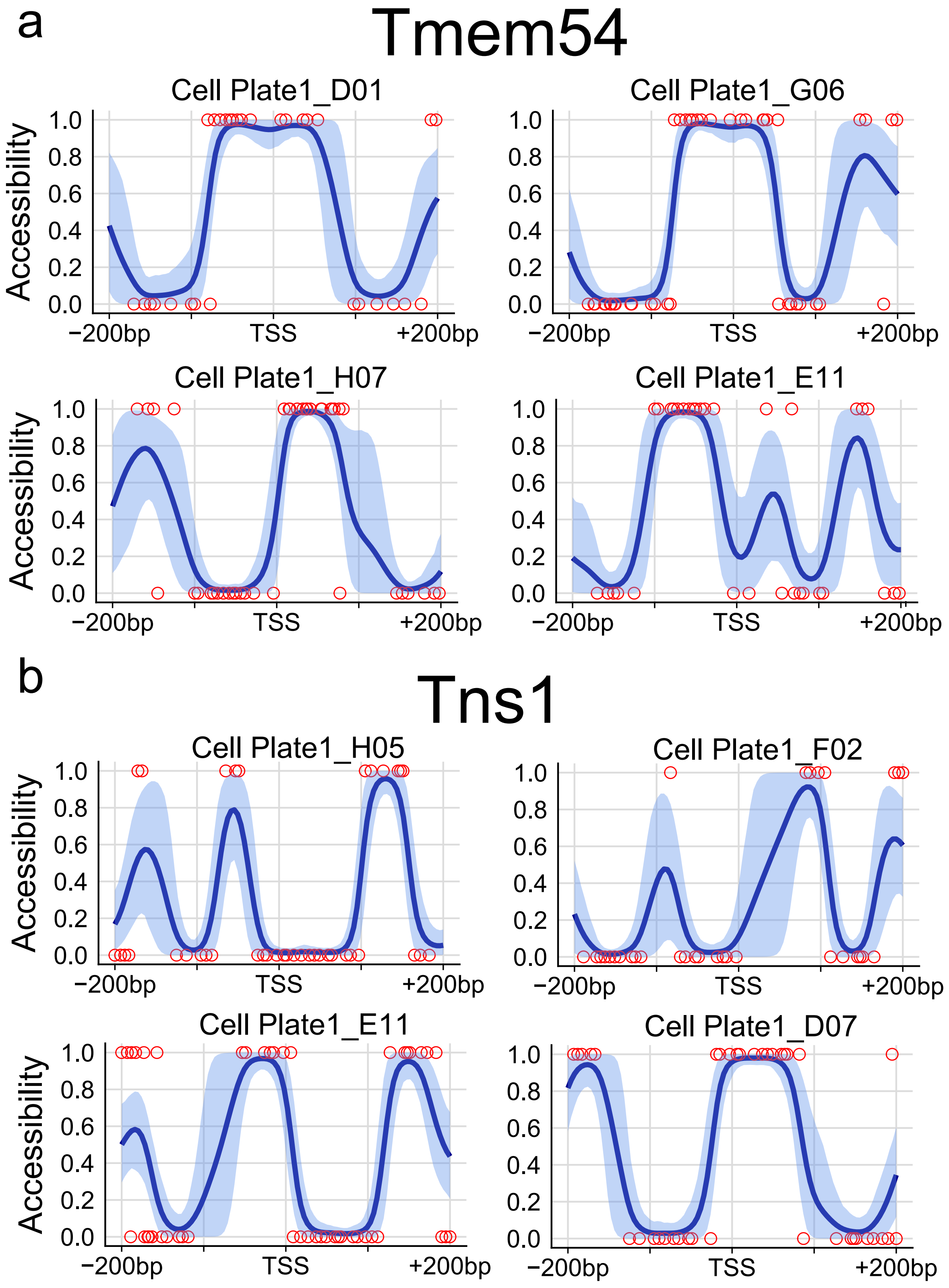

Supplementary figure 14. Example of single-cell accessibility profiles at transcription start sites. Shown are profiles generated from four arbitrary cells in two example genes, (a) *Tmem54* and (b) *Tns1*. Each red dot represents a GpC site, with binary accessibility value (1=accessible, 0=inaccessible). Blue line represents the mean of the posterior distribution of the inferred non-linear function, and the shading represents the corresponding 80% credible interval. Inference was done using the BPRMeth package[5]. Axis ticks display windows of +-200bp around the TSS. We observe periodic patterns in the GC accessibility data, which likely indicate positions of nucleosomes

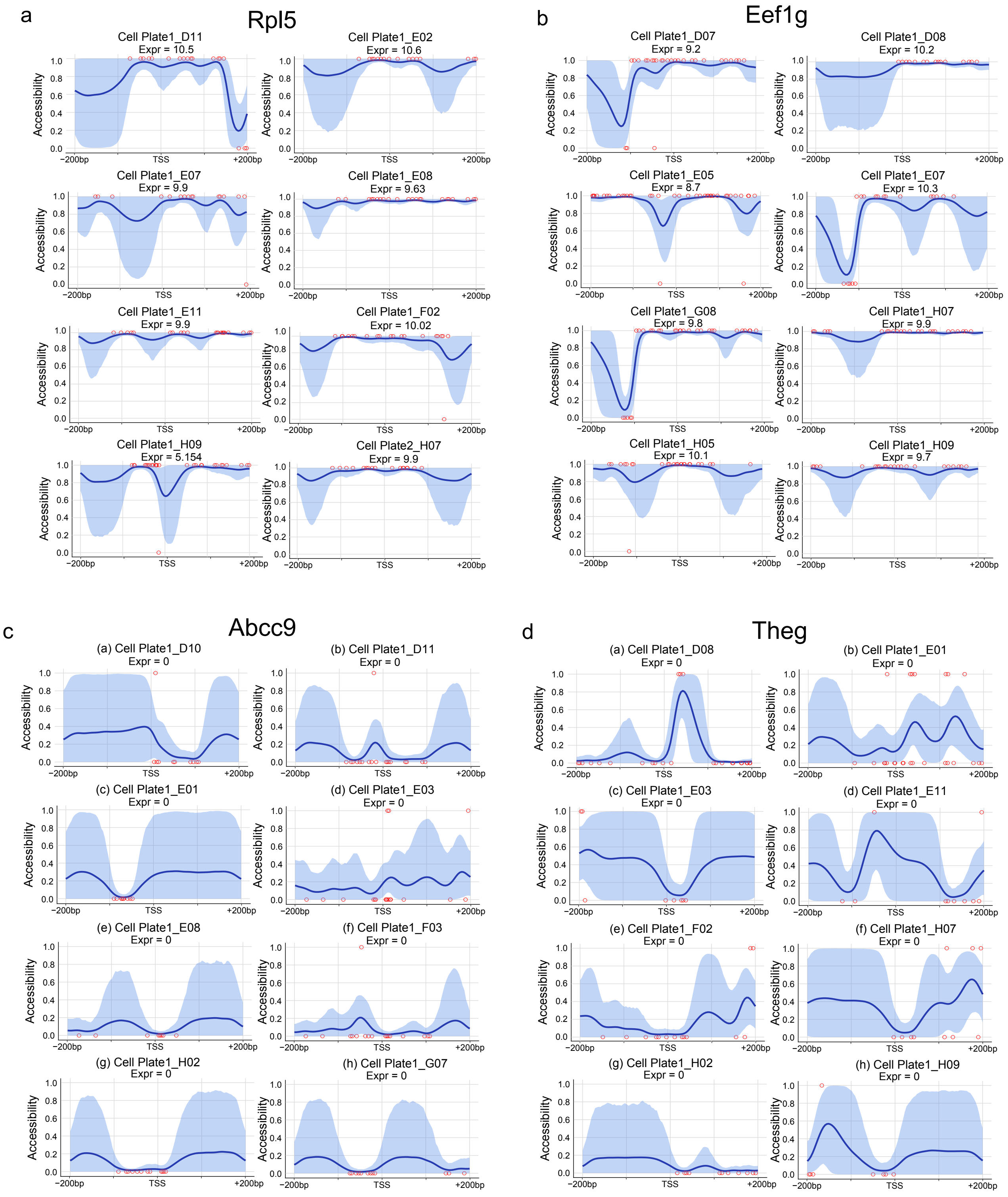

Supplementary figure 15. Reconstructed single-cell accessibility profiles from genes with different RNA expression regimes. Shown are profiles of representative cells for highly accessible and expressed housekeeping genes: (a) Rpl5 and (b) *Eef1g*, and for non-accessible and non-expressed genes: (c) *Abcc9* and (d) *Theg*. Each red dot represents a GpC site, with binary accessibility value (1=accessible, 0=inaccessible). A non-linear regression curve was fit for each gene and cell using the BPRMeth package[5]. The blue line represents the mean of the posterior distribution of the inferred non-linear function, and the shading represents the corresponding 80% credible interval. Axis ticks display windows of +-200bp around the TSS.

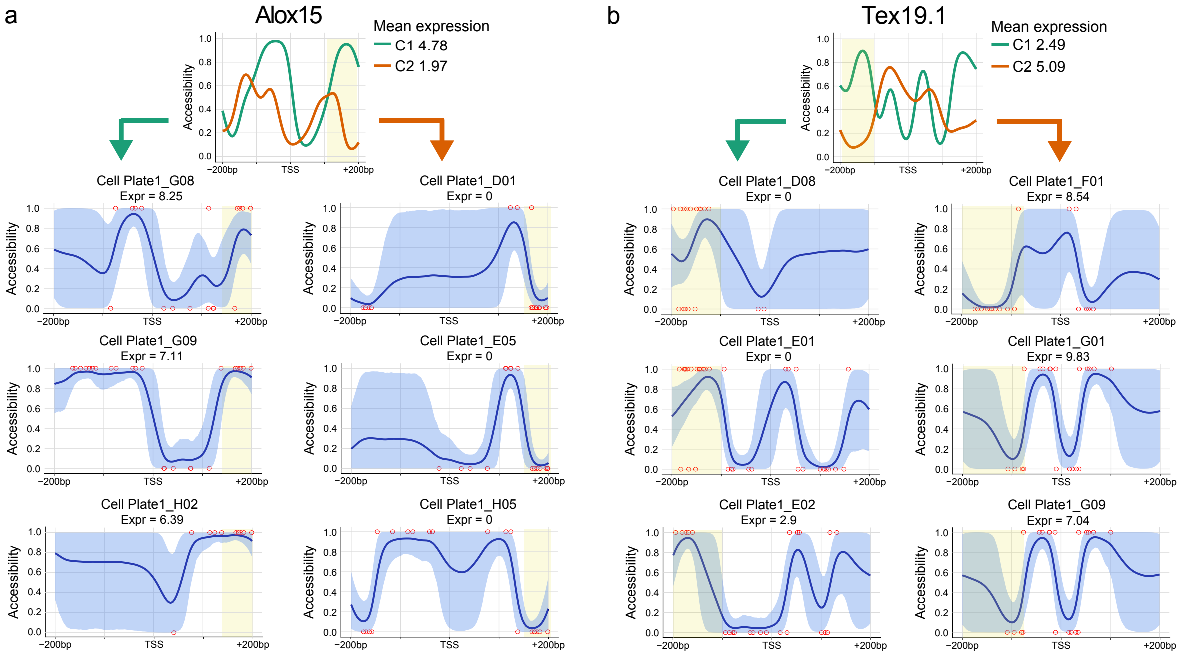

Supplementary figure 16. Example of GpC accessibility profiles at genes with K=2 clusters associated with differential gene expression Shown are accessibility profiles for two representative genes with K=2 clusters that display cluster-driven changes in gene expression: (a) Alox15 and (b) Tex19.1. The average pseudo-bulked profiles per gene and cluster (green and orange lines) are represented at the top, together with the corresponding average RNA expression levels. Representative examples of the single-cell profiles are shown at the bottom. Each red dot represents a GpC site, with binary accessibility value (1=accessible, 0=inaccessible). A non-linear regression curve was fit for each gene and cell using the BPRMeth package[5]. The blue line represents the mean of the posterior distribution of the inferred non-linear function, and the shading represents the corresponding 80% credible interval. Axis ticks display windows of +-200bp around the TSS.

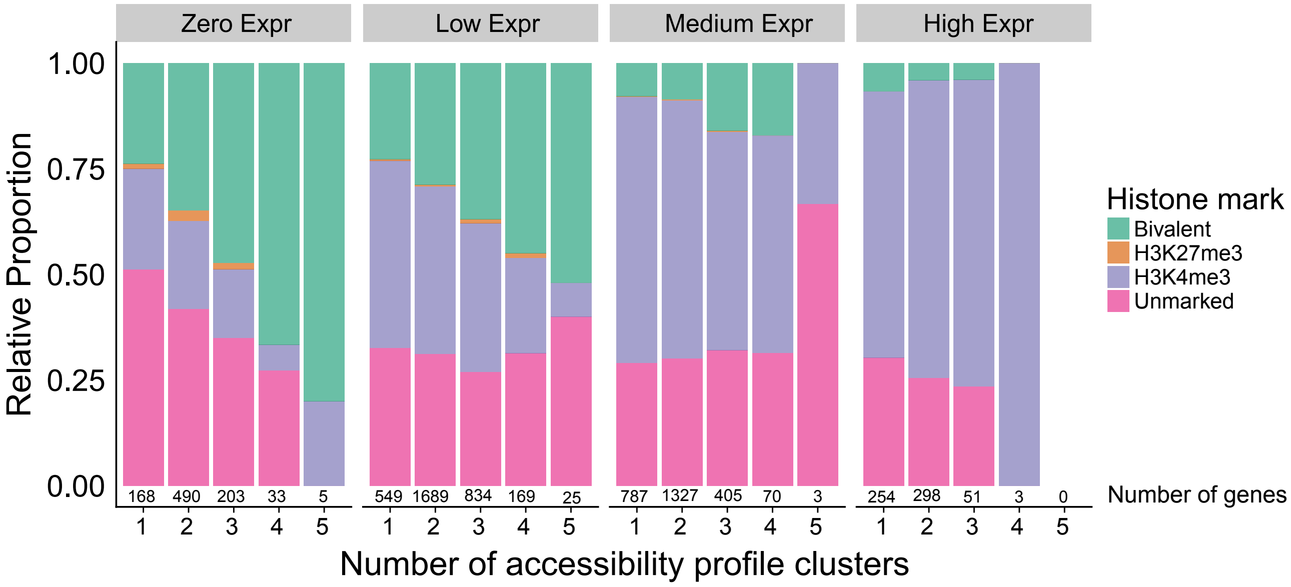

Supplementary Figure 17. Presence of bivalent histone marks (H3K4me3 and H3K27me3) is associated with high cell-to-cell variability in accessibility profiles. For each gene, a measure of heterogeneity in the accessibility profiles was computed by clustering all cells and learning the most likely number of clusters. Genes with a single cluster (K=1) correspond to a more homogeneous chromatin pattern than genes with multiple clusters. The results were overlapped with ChiP-seq histone marks data. The number of clusters (i.e. heterogeneity) is displayed in the x axis, and the relative proportion of each histone mark is displayed in the y axis. To account for differences in mean expression levels, genes were split in four different expression groups (“Zero Expr” for an average log normalised counts equal to 0, “Low Expr” between 0 and 2, “Medium Expr” between 2 and 6 and “High Expr” higher than 6)

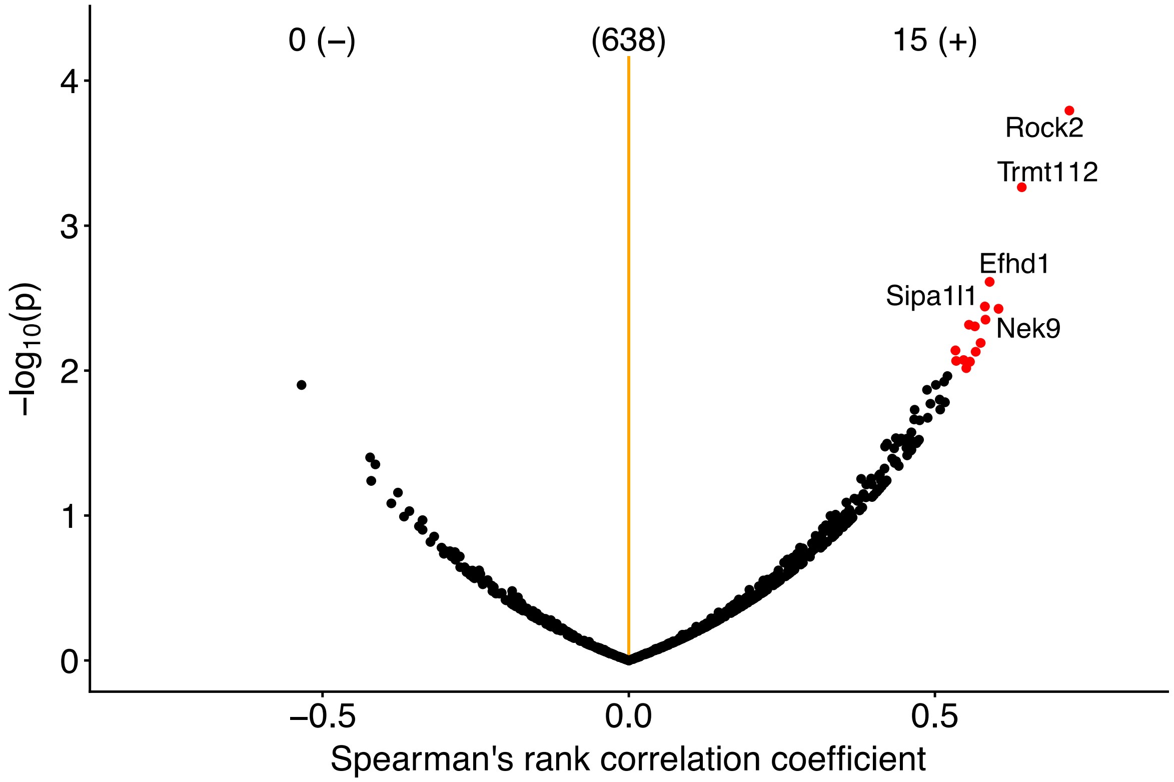

Supplementary Figure 18. Association analysis between promoter accessibility profile and development trajectory. For each gene, the cell cluster assignments were associated with the corresponding cell's position in the pseudotime axis using Spearman's rank coefficient. Shown is a volcano plot of correlation coefficients in the x axis with the corresponding log10 p-values in the y axis. Red dots denote genes that pass statistical significance threshold (alpha = 0.01).

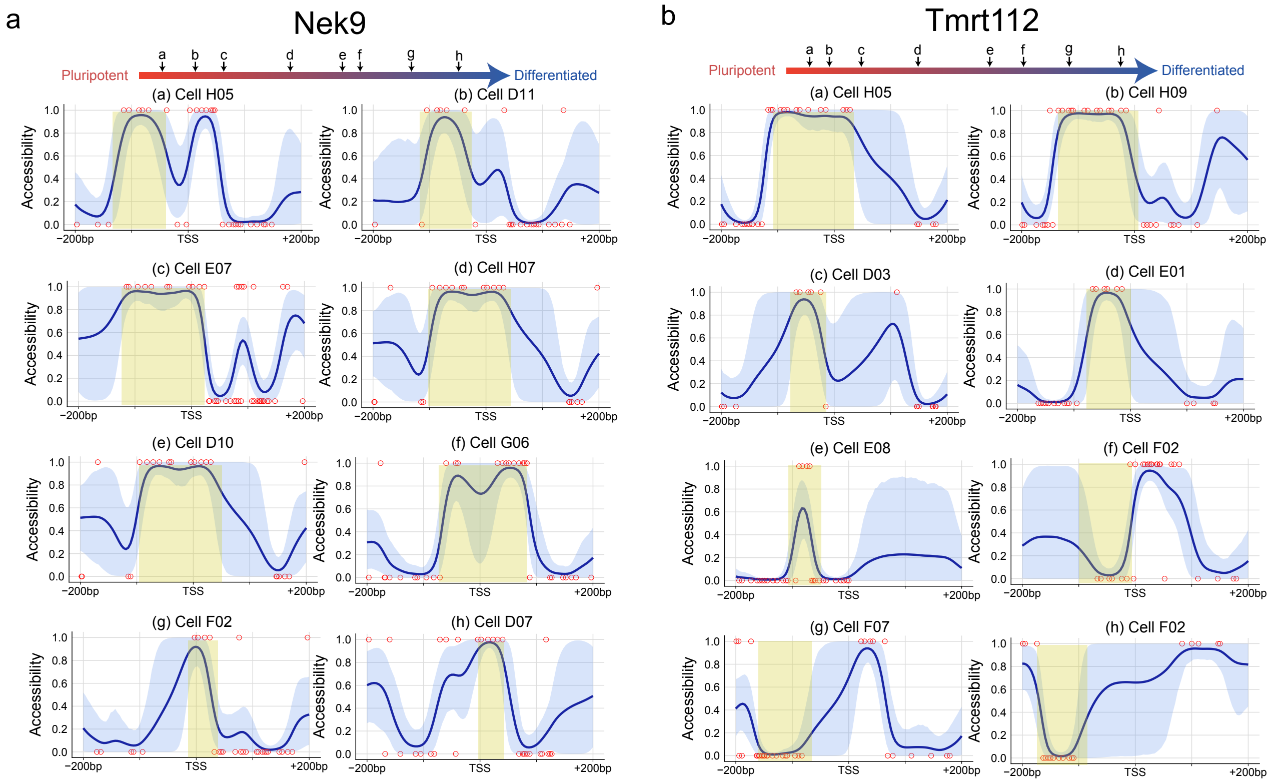

Supplementary figure 19. Reconstructed dynamics of chromatin accessibility profiles along the developmental trajectory. Shown are profiles of representative cells for genes that show dynamic behaviour along the pseudotime in their accessibility profile: (a) *Nek9* and (b) *Trmt112*. Each red dot represents a GpC site, with binary accessibility value (1=accessible, 0=inaccessible). A non-linear regression curve is fit for each gene and cell using the BPRMeth package[5]. The blue line represents the mean of the posterior distribution of the inferred non-linear function, and the shading represents the corresponding 80% credible interval. Axis ticks display windows of +-200bp around the TSS. Yellow shading is used to highlight the relevant region of dynamic changes.

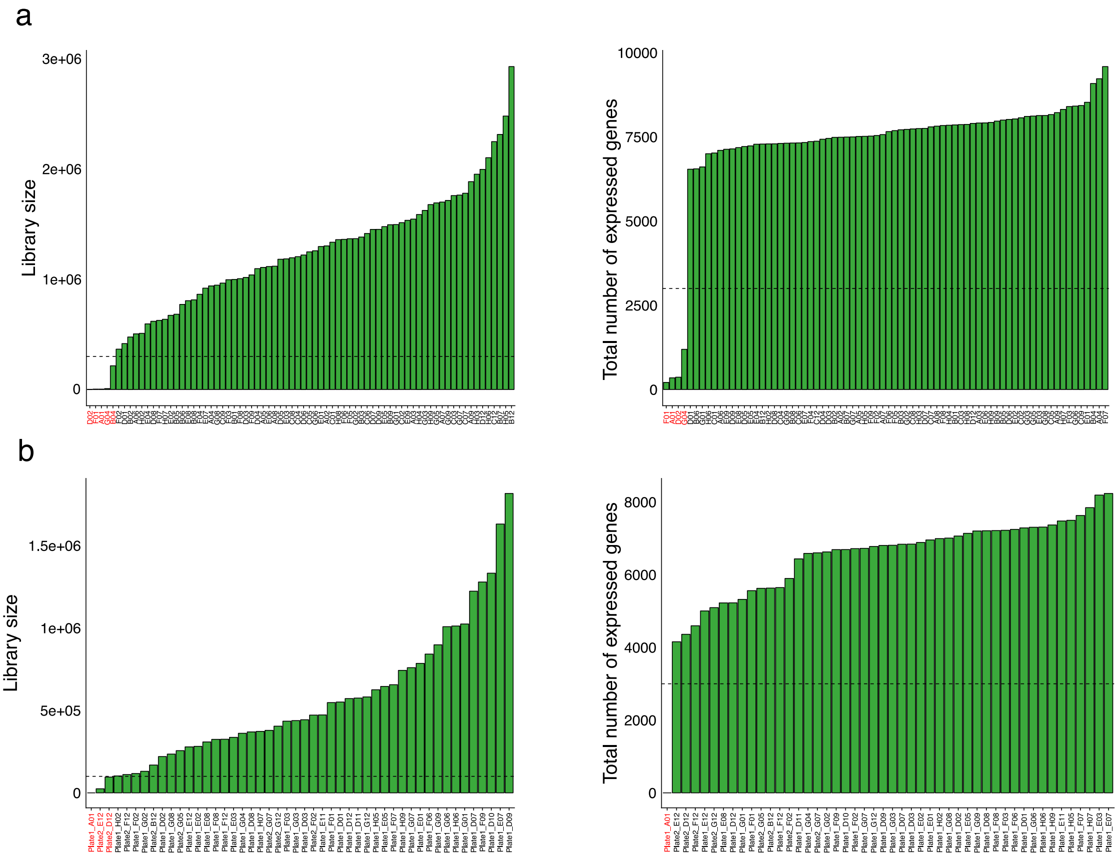

Supplementary figure 20. RNA-seq quality control for (a) ESC and (b) EB dataset. Left displays the number of aligned reads per cell (library size) and right is the number of expressed genes (log2 normalised read counts>0) detected per cell. Cells below a set threshold (dotted lines) were removed (axis text in red).

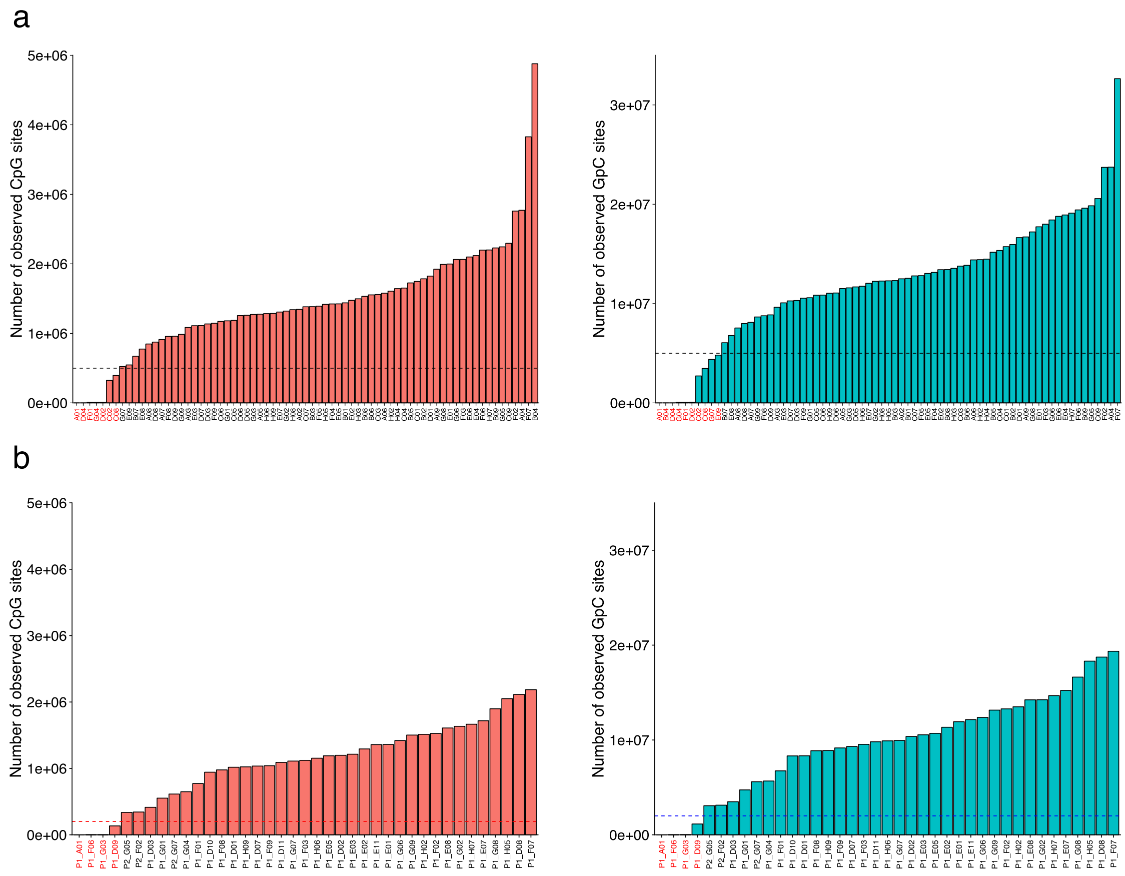

Supplementary figure 21. BS-seq quality control for (a) ESC and (b) EB dataset. Displayed are the number of observed cytosine’s in either CpG (left) or GpC (right) context. Cells below a set threshold (dotted lines) were removed (axis text in red).

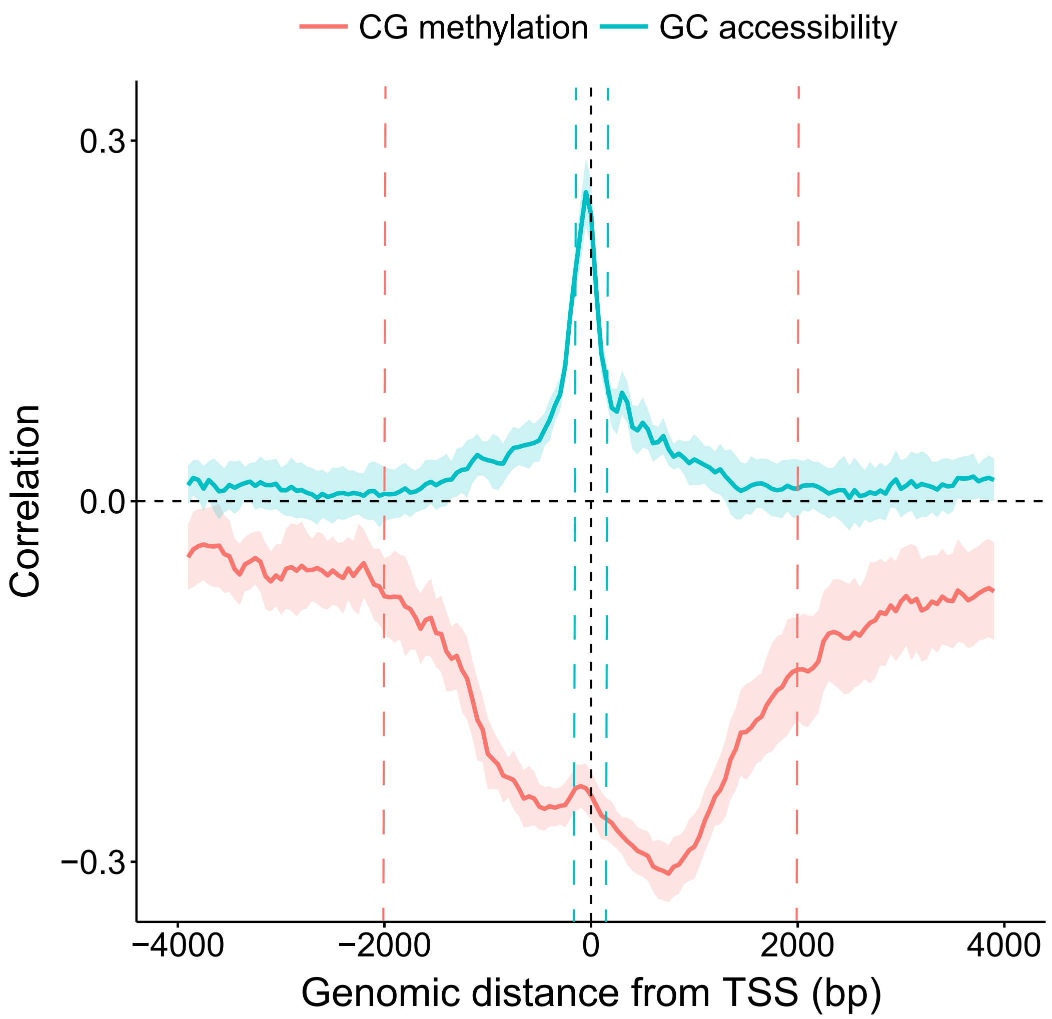

Supplementary figure 22. Defining windows for correlation analysis in promoter regions. Pearson correlation coefficients between accessibility and transcription (blue) and methylation and transcription (red) in 100bp sliding windows. Solid line shows the mean across all genes and cells and the shade shows the corresponding standard deviation. The dashed lines display the selected windows for the correlation analysis: +-50bp and +-2000 bp around the TSS in accessibility and methylation, respectively.

Supplementary References

1. Angermueller, C. et al. *Nat Meth* **13**, 229-232 (2016).
2. Pierson, E. & Yau, C. *Genome Biology* **16**, 241 (2015).
3. Kolodziejczyk, Aleksandra A. et al. *Cell Stem Cell* **17**, 471-485 (2015).
4. Zvetkova, I. et al. *Nat Genet* **37**, 1274-1279 (2005).
5. Kapourani, C.A. & Sanguinetti, G. *Bioinformatics (Oxford, England)* **32**, i405-i412 (2016).
