# Supplementary material for: scNMT-seq enables joint profiling of chromatin accessibility DNA methylation and transcription in single cells
Source: Nat Commun. 2018 Feb 22;9:781. doi: 10.1038/s41467-018-03149-4 (PMC5823944; doi:10.1038/s41467-018-03149-4)
Supplement: Supplementary file 3 — Description of Additional Supplementary Files [file 41467_2018_3149_MOESM3_ESM.pdf]

Description of Additional Supplementary Files

File Name: Supplementary Data 1
Description: Sequencing quality metrics for scBS-seq and scRNA-seq libraries.

File Name: Supplementary Data 2
Description: Summary statistics obtained for the cell-specific association analysis correlating methylation with expression, accessibility with expression or methylation with accessibility at individual loci.

File Name: Supplementary Data 3
Description: Genomic annotations used in this study.
